# Epidemiology of seasonal influenza in the Middle East and North Africa regions, 2010‐2016: Circulating influenza A and B viruses and spatial timing of epidemics

**DOI:** 10.1111/irv.12544

**Published:** 2018-02-19

**Authors:** Saverio Caini, Clotilde El‐Guerche Séblain, Meral A. Ciblak, John Paget

**Affiliations:** ^1^ Netherlands Institute for Health Services Research (NIVEL) Utrecht The Netherlands; ^2^ Global Health Economics and Outcomes Research (HEOR) Influenza Lead Sanofi Pasteur Lyon France; ^3^ Regional Influenza Expert, Africa/Eurasia and Middle East region Sanofi Pasteur Istanbul Turkey

**Keywords:** influenza type A, influenza type B, Middle East, North Africa, timing of epidemics

## Abstract

**Background:**

There is a limited knowledge regarding the epidemiology of influenza in Middle East and North Africa.

**Objectives:**

We described the patterns of influenza circulation and the timing of seasonal epidemics in countries of Middle East and North Africa.

**Methods:**

We used virological surveillance data for 2010‐2016 from the WHO FluNet database. In each country, we calculated the median proportion of cases that were caused by each virus type and subtype; determined the timing and amplitude of the primary and secondary peaks; and used linear regression models to test for spatial trends in the timing of epidemics.

**Results:**

We included 70 532 influenza cases from seventeen countries. Influenza A and B accounted for a median 76.5% and 23.5% of cases in a season and were the dominant type in 86.8% and 13.2% of seasons. The proportion of influenza A cases that were subtyped was 85.9%, while only 4.4% of influenza B cases were characterized. For most countries, influenza seasonality was similar to the Northern Hemisphere, with a single large peak between January and March; exceptions were the countries in the Arabian Peninsula and Jordan, all of which showed clear secondary peaks, and some countries had an earlier primary peak (in November‐December in Bahrain and Qatar). The direction of the timing of influenza activity was east to west and south to north in 2012‐2013 and 2015‐2016, and west to east in 2014‐2015.

**Conclusions:**

The epidemiology of influenza is generally uniform in countries of Middle East and North Africa, with influenza B playing an important role in the seasonal disease burden.

## 1 INTRODUCTION

The epidemiology of seasonal influenza is well defined in many regions of the world, particularly in developed countries of the Northern and Southern Hemispheres. In other regions of the world, much less is known about the epidemiology of influenza A and B, especially in the tropics and subtropics^1^. The number of publications related to the epidemiology of influenza in low‐income countries^2^, ^3^, ^4^, including regional^5^, ^6^, ^7^ and global analyses^8^, ^9^, ^10^, has been growing in recent years. This information is important as it can be used by public health authorities and national healthcare systems to define and manage country‐specific prevention and control programmes for seasonal influenza (eg to define the optimal time to launch a vaccination campaign).

This study evaluates the epidemiology of seasonal influenza A and B in the Middle East and North Africa. These regions separate the tropical countries of Africa and Asia in the south from Europe, Russia and Central Asia in the North, and have a predominantly subtropical climate. Currently, annual vaccination campaigns focusing on the elderly and specific risk groups (including children, healthcare workers and Hajj pilgrims) are being recommended in most countries of the Middle East and North Africa^11^; however, the number of doses distributed per 1000 population is still low and more efforts are needed to increase the vaccine uptake in this region^12^. A better knowledge of influenza epidemiology and patterns of spread will provide further support to these campaigns.

Several reports have appeared in recent years that have described the epidemiology of influenza and disease burden in single countries of this region^13^, ^14^, ^15^, ^16^, ^17^; some others have tried to assess the impact of the annual Hajj pilgrimage to Saudi Arabia on the transmission of viral and bacterial respiratory infections, including influenza^18^, ^19^. However, no unified, comparative approach has been attempted to study the epidemiology of seasonal influenza in the Middle East and North Africa. The aim of our study was to compare countries in this region in terms of the circulating influenza A and B viruses and to assess the spatial timing of seasonal epidemics in this area.

## 2 MATERIALS AND METHODS

**Figure 1:**
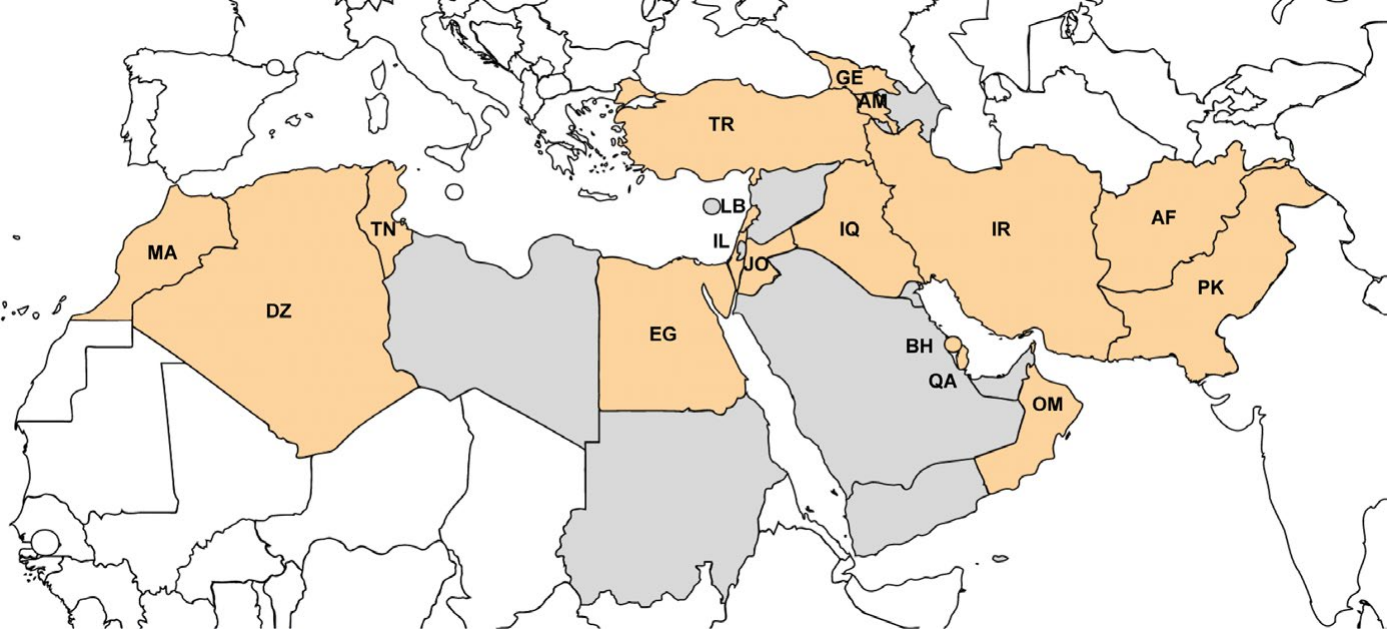
Countries included in the analysis (in red) and countries that were not included because of lack of data (in grey). Countries were marked using the two‐letter ISO codes. AF, Afghanistan; AM, Armenia; BH, Bahrain; DZ, Algeria; EG, Egypt; GE, Georgia; IL, Israel; IQ, Iraq; IR, Iran; JO, Jordan; LB, Lebanon; MA, Morocco; OM, Oman; PK, Pakistan; QA, Qatar; TN, Tunisia; TR, Turkey

Our analysis focused on a large world region encompassing twenty‐seven countries in the Middle East and North Africa (Figure 1). These countries cover a combined population of nearly 800 million inhabitants.

### 2.1 Source of data

We took advantage of influenza surveillance data contained in the FluNet database for our analysis. FluNet is a publicly available, Web‐based database that has been operated by the WHO Global Influenza Surveillance and Response System (GISRS) since 1995^20^. Epidemiological and virological influenza surveillance data are entered into the FluNet database by collaborating National Influenza Centres and other WHO influenza reference laboratories around the world. The FluNet database contains the weekly number of respiratory specimens that were collected and processed in each country, and the number of those that tested positive for influenza type A and B, A subtype (AH1, A[H1N1]pdm2009, AH3, AH5, not subtyped) and B lineage (Victoria, Yamagata, not determined).

A preliminary check of the FluNet database revealed that most countries in North Africa and the Middle East had no or very little influenza surveillance data before the 2009 pandemic. Therefore, we opted to limit the study period to the post‐pandemic period, in order to ensure greater comparability of information between countries.

Influenza epidemics usually occur during winter months and can bridge consecutive calendar years in the Northern Hemisphere. Therefore, we used as unit of analysis the “country season,” defined as the period between 1 July of 1 year and 30 June of next year. On 12 November 2016, we downloaded influenza surveillance data available from all countries of interest (Figure 1) for the period between 1 July 2010 and 30 June 2016, that is for the six consecutive seasons from 2010‐2011 to 2015‐2016.

### 2.2 Statistical analysis

For each country, the statistical analysis was limited to seasons with 50 or more influenza reported cases and at least 20 weeks of data reporting in order to enhance the reliability of results^9^. We determined the proportion of influenza cases that were caused by each virus type, subtype and lineage in each country and season, and determined the median values for each country (ie across seasons) and for the whole region (ie across all seasons and countries). Medians were compared between groups of countries using the Mann‐Whitney nonparametric test.

We aimed to determine the typical timing and seasonality of the primary and secondary epidemic peaks in each country using the epipoi software^21^. The epipoi method first divides the weekly number of reported influenza cases by the maximum value in the same season: this is done to standardize all season‐specific time series in each country, so that all seasons are given the same weight in subsequent analysis. It then models the trend and seasonality of each country‐specific influenza time series by summing annual, semi‐annual and quarterly harmonics (obtained from Fourier decomposition): the curve generated in this way is named the periodic annual function (PAF)^21^, ^22^. The timing of the primary peak refers to the month when influenza activity (as modelled by the PAF) reaches its maximum value; if the PAF is a bimodal curve, a secondary peak is also identified. The typical seasonality of influenza epidemics is quantified by calculating the amplitude of the primary and secondary peaks: this is defined as the ratio of the wave height (difference between the peak and the trough) in the PAF and the peak value, and it is an estimate of the degree to which influenza cases in each season tend to cluster around the typical peak timing extracted from the PAF (when the trough value in the model is below zero, the amplitude can exceed 100%). The peak and amplitude of the primary and secondary peaks were calculated for all influenza cases and separately for influenza A and B epidemics (for the latter analysis, for each country we only included seasons with 50 or more influenza cases caused by that virus type). For more details on how the PAF is fitted to the raw data and the timing and amplitude of the peaks are calculated using the epipoi software, the reader can refer to the paper by Alonso and McCormick where the software is presented^21^.

Finally, we aimed to detect whether seasonal influenza epidemics spread along geographical gradients in countries of the Middle East and North Africa. For this purpose, we fitted a linear regression model for each season using the longitude and latitude of the country's centroid as independent variables, and the timing of the typical (ie obtained from the PAF) primary epidemic peak (with weeks numbered consecutively from July of 1 year to June of next year) as the dependent variable^23^. A similar analysis was conducted in parallel also for Europe and Central Asia (ie countries included in the south‐west Europe, northern Europe, eastern Europe and Central Asia Influenza Transmission Zones^24^) to test whether the spatial timing of influenza epidemics in the same season is comparable in these two world areas (the Middle East and North Africa vs Europe and Central Asia).

Analyses were conducted using Stata version 14 (Stata Corp, College Station, TX) and epipoi 21. Maps were obtained using freely available software (http://mapchart.net/). All tests were two‐sided and considered as statistically significant when the *P*‐value was .05 or less.

## 3 RESULTS

A total of 70 915 laboratory‐confirmed influenza cases from nineteen countries in the Middle East and North Africa between 1 July 2011 and 30 June 2016 were contained in the FluNet database. After applying the exclusion criteria, 70 532 cases from 83 seasons in seventeen countries were included in the analysis (there were no seasons with 50 or more reported influenza cases in Azerbaijan and Syria) (Figure 1). The number of seasons per country that were included in the analysis ranged from one (2014‐2015 in Armenia and 2015‐2016 in Afghanistan) to six (eleven countries) (Table 1). Influenza cases were unevenly distributed across countries, and over 50% of overall cases were contributed by only three countries (Turkey, Qatar and Egypt). The median number of influenza cases per season and country was 440 (interquartile range [IQR]: 202 – 994). The number of influenza cases that was caused by each virus type, subtype and lineage in each country and season included in the analysis is reported in Table S1.

**Table 1:** Laboratory‐confirmed influenza samples reported in each country by virus type, subtype and lineage. The WHO FluNet database, 2010‐2016

Country	Population (million)	Latitude a	Longitude a	No. of seasons^b^	Influenza (any type)	A(H3N2)	A(H1N1)pdm2009	A other/not subtyped	B Victoria	B Yamagata	B not characterized	Median % A (min, max)	Median % B (min, max)
Afghanistan	33.3	33° N	65° E	1	74	13	6	49	0	0	6	91.9 (‐)	8.1 (‐)
Algeria	40.3	28° N	3° E	6	1832	503	709	2	16	24	578	82.5 (45.0‐99.5)	17.5 (0.5‐55.0)
Armenia	3.1	40° N	45° E	1	632	0	627	5	0	0	0	100.0 (‐)	0.0 (‐)
Bahrain	1.4	26° N	50° E	4	1022	86	694	74	17	59	92	76.3 (71.4‐88.8)	23.7 (11.2‐28.6)
Egypt	94.7	27° N	30° E	6	9997	2046	5297	449	10	11	2184	73.8 (62.0‐96.4)	26.2 (3.6‐38.0)
Georgia	4.9	42° N	43° E	6	1894	305	932	15	13	0	629	80.6 (14.9‐96.1)	19.4 (3.9‐85.1)
Iran	82.8	32° N	53° E	6	8315	1893	4184	4	0	0	2234	67.5 (44.0‐90.5)	32.5 (9.5‐56.0)
Iraq	38.1	33° N	44° E	4	1672	36	1190	371	0	0	75	98.2 (77.1‐100.0)	1.8 (0.0‐22.9)
Israel	8.2	31° N	34° E	6	7081	1168	2213	1793	0	0	1907	72.7 (53.6‐93.2)	27.3 (6.8‐46.4)
Jordan	8.2	31° N	36° E	6	1685	382	929	4	97	61	212	86.3 (38.5‐99.1)	13.7 (0.9‐61.5)
Lebanon	6.2	33° N	35° E	2	302	38	76	0	0	0	188	35.4 (21.6‐49.2)	64.6 (50.8‐78.4)
Morocco	33.7	32° N	5° W	5	1178	370	412	2	28	34	332	60.4 (32.7‐94.8)	39.6 (5.2‐67.3)
Oman	3.4	21° N	57° E	6	5021	949	2186	477	0	76	1333	73.4 (60.2‐90.1)	26.6 (9.9‐39.8)
Pakistan	202.0	30° N	70° E	6	2273	369	964	466	66	73	335	77.6 (50.9‐89.9)	22.4 (10.1‐49.1)
Qatar	2.3	25° N	51° E	6	11 581	84	5220	3815	0	1	2461	77.7 (65.4‐86.8)	22.3 (13.2‐34.6)
Tunisia	11.1	34° N	9° E	6	1462	381	706	1	0	77	297	80.4 (56.0‐95.9)	19.6 (4.1‐44.0)
Turkey	80.3	39° N	35° E	6	14 511	4225	6792	40	89	2	3363	76.0 (49.2‐97.2)	24.0 (2.8‐50.8)
Total	654.0	21° N ‐ 42° N	5° W ‐ 70° E	83	70 532	12 848	33 137	7567	336	418	16 226	76.5 (14.9‐100.0)	23.5 (0.0‐85.1)

a Latitude and longitude of the country centroid (if available) or largest city.

b A season was defined as the period between 1 July of one year and 30 June of next year. Only seasons with 50 or more reported influenza cases and 20 or more weeks of data reporting were included. See text for more details.

Influenza A and B accounted for a median 76.5% (IQR 60.4%‐90.3%) and 23.5% (IQR 9.7%‐39.6%) of all influenza cases in a season during the study period, respectively (Table 1). More specifically, influenza B accounted for fewer than 20% of reported influenza cases in 34 of the 83 seasons (41.0%) between 20% and 50% in 38 seasons (45.8%), between 50% and 80% in 10 seasons (12.0%) and above 80% in one season (1.2%). There were slight, non‐significant differences (*P*‐value = .96) in the median proportion of influenza cases that were caused by type B virus in the Middle East (23.2%) and North Africa (27.3%).

The largest proportion of influenza A cases was caused by the pandemic A(H1N1) strain (61.9%), followed by the A(H3N2) subtype (24.0%); the remaining influenza A cases were either not subtyped (14.1%) or due to the pre‐pandemic A(H1N1) strain (<0.1%). Among all influenza A cases that were subtyped, the median proportion of those that were caused by the A(H1N1) and A(H3N2) subtypes did not differ significantly (*P* = .166) between countries of the Middle East (68.4% and 31.6%) and North Africa (65.9% and 34.1%). The proportion of influenza B cases that were characterized was above 20% in only four countries (45.2% in Bahrain, 42.7% in Jordan, 29.3% in Pakistan and 20.6% in Tunisia): in these countries, Victoria and Yamagata accounted for 40.0% and 60.0% of influenza B cases for which the lineage was determined, respectively. The lineage was unknown for all influenza B cases from six countries (Afghanistan, Iran, Iraq, Israel, Lebanon and Qatar) (Table 1).

### 3.1 Timing of influenza epidemics

**Figure 2:**
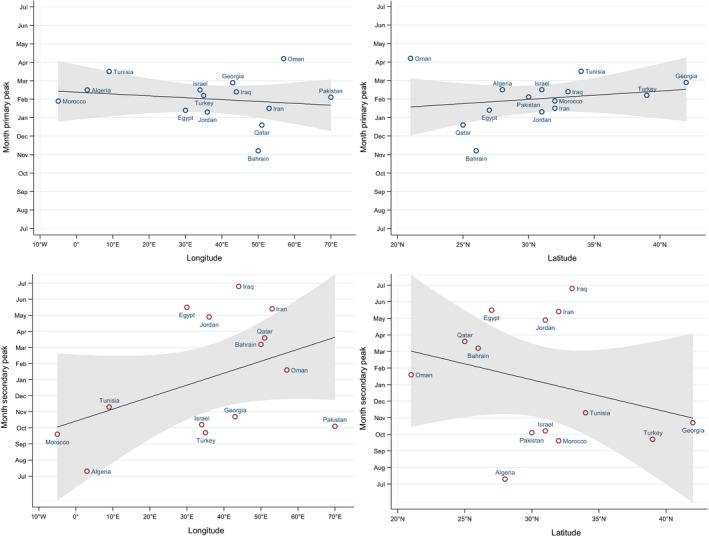
Typical timing of the primary and secondary peaks of influenza detection (as calculated using the periodic annual function; see text for details) by site, against their longitudinal and latitudinal positions

The inspection of the typical timing and amplitude of the primary and secondary peaks of influenza epidemics (Table 2 and Figure 2; the standardized time series and PAF for each country included in the analysis are available in the Figure S1) revealed the following prominent characteristics:

**Table 2:** Timing and amplitude of the primary and secondary peaks of seasonal influenza epidemics (only countries with four or more seasons of data were included). The WHO FluNet database, 2010‐2016

Country	Latitude^a^	Longitude^a^	Primary peak		Secondary peak	
			Timing (Month)	Amplitude	Timing (Month)	Amplitude
Algeria	28° N	3° E	Feb	109.7%	Jul	15.6%
Bahrain	26° N	50° E	Nov	123.6%	Mar	83.3%
Egypt	27° N	30° E	Jan	102.1%	May	27.0%
Georgia	42° N	43° E	Feb	109.9%	Oct	27.7%
Iran	32° N	53° E	Jan	112.0%	May	26.8%
Iraq	33° N	44° E	Feb	115.3%	Jun	26.8%
Israel	31° N	34° E	Feb	105.1%	Oct	12.3%
Jordan	31° N	36° E	Jan	113.0%	Apr	105.1%
Morocco	32° N	5° W	Jan	104.0%	Sep	12.1%
Oman	21° N	57° E	Apr	83.0%	Jan	82.4%
Pakistan	30° N	70° E	Feb	103.5%	Oct	25.5%
Qatar	25° N	51° E	Dec	106.5%	Mar	76.0%
Tunisia	34° N	9° E	Mar	108.6%	Nov	18.2%
Turkey	39° N	35° E	Feb	112.7%	Sep	22.7%

a Latitude and longitude of the country centroid (if available) or largest city.

- All countries except those situated in the Arabian peninsula had well‐defined primary peaks taking place typically in January‐March, and much smaller secondary peaks placed either in spring/summer (May to July) or in autumn (September to November), with the sole exception being Jordan (whose secondary peak was typically in April and had an amplitude only slightly less than that of the primary peak);
- Bahrain and Qatar (two smaller countries on the east coast of the Arabian peninsula, situated at a short distance from each other) were characterized by having a very clear primary peak in November‐December and a secondary peak of sizeable amplitude (83.3% in Bahrain, 76.0% in Qatar) in March;
- In Oman (the southernmost country among those included in the analysis; it is situated in the Arabian Peninsula and overlooks the Indian Ocean), the primary and secondary peaks had only slightly different amplitude (83.0% and 82.4%, respectively); the former typically took place in March and the latter in January.

The epidemics caused by influenza A and B tended to peak within 2 weeks of each other in eight countries (Georgia, Iran, Iraq, Israel, Morocco, Pakistan, Tunisia and Turkey) (results not shown). Influenza A epidemics typically peaked two or more weeks before influenza B in five countries (Bahrain, Egypt, Jordan, Qatar and Turkey), while the opposite occurred in Algeria and Oman (results not shown).

### 3.2 Spatial timing of influenza epidemics

The analysis of spatial spread of epidemics (Table 3) revealed that there were significant or nearly significant geographical gradients according to the country longitude only in half of the seasons in the Middle East and North Africa region: in the east‐to‐west direction in 2012‐2013 and 2015‐2016, and in the west‐to‐east direction in 2014‐2015. In general, the geographical gradients of the timing of influenza epidemics did not coincide with those observed in Europe and Central Asia, except for the east‐to‐west gradient in 2015‐2016 and the lack of any gradient in 2011‐2012. Also, we detected a south‐to‐north latitudinal gradient in the 2012‐2013 and 2015‐2016 seasons in the Middle East and North Africa region, while a latitudinal gradient (also in the south‐to‐north direction) was observed in Europe and Central Asia only in 2013‐2014. As the geographical gradient (by longitude and/or latitude) of influenza epidemic peaks was detected only in a few seasons and could be in opposite direction, there was no statistically significant association between geographical coordinates and the typical timing of the primary peak as calculated from the PAF.

**Table 3:** Comparison of the spatial timing of influenza epidemics in Middle East and North Africa and in Europe and Central Asia in six consecutive influenza seasons (from 2010‐2011 to 2015‐2016). The WHO FluNet database, 2010‐2016

Season^a^	Longitudinal gradient				Latitudinal gradient			
	Middle East and North Africa	*P*‐value	Europe and Central Asia^b^	*P*‐value	Middle East and North Africa	*P*‐value	Europe and Central Asia^b^	*P*‐value
2010‐2011	‐	.407	West to east	.008	‐	.241	‐	.576
2011‐2012	‐	.846	‐	.362	‐	.615	‐	.236
2012‐2013	East to west	.035	‐	.443	South to north	.027	‐	.111
2013‐2014	‐	.486	West to east	.089	‐	.121	South to north	.048
2014‐2015	West to east	.061	‐	.399	‐	.909	‐	.567
2015‐2016	East to west	.064	East to west	.002	South to north	.092	‐	.716

a A season was defined as the period between 1 July of one year and 30 June of next year.

b This includes all countries in the following influenza transmission zones: south‐west Europe, northern Europe, eastern Europe and Central Asia (see [WHO Influenza Transmission Zones]).

## 4 DISCUSSION

We compared the relative contribution of the different virus types and subtypes to influenza epidemics and investigated the spatio‐temporal patterns of seasonal influenza activity in seventeen countries of the Middle East and North Africa during six consecutive seasons (2010‐2011 to 2015‐2016). Influenza A was dominant in most seasons, with the pandemic strain accounting for the majority of cases. On average, influenza B caused nearly one‐fourth of all cases in a season and was dominant in roughly one of seven seasons, which is in line with the pattern observed in other world areas^9^ and confirms the role of influenza B as an important contributor to the seasonal influenza burden.

The seasonality of influenza epidemics was in line with the Northern Hemisphere for most countries in the region, with large primary peaks between January and March, and no or nearly undetectable secondary peaks. The most notable exceptions were Jordan (which showed a secondary peak in April) and the three countries located in the south‐eastern part of the Arabian Peninsula, with Bahrain and Qatar having earlier primary peaks (in November and December) and a very clear secondary peak in March, while Oman having two peaks with very similar amplitudes in January and April. Bahrain, Qatar and Oman were the southernmost countries included in our analysis; in fact, they are situated at roughly the same latitudes as countries which are characterized by less defined influenza seasonality and multiple epidemic peaks, such as India^25^ and southern China^7^. Climatic parameters, such as absolute humidity, temperature and rainfalls, shape influenza seasonality, both in temperate and tropical climate countries^26^, and are likely to be the main driver of influenza seasonality in the Middle East and North Africa as well, although only a few studies have been conducted so far in this region^27^. Population movements (eg annual Hajj pilgrimage, migrants and nomadic populations) might also contribute to determine influenza seasonality in some countries and the spread of epidemics across the region. Despite the observed differences between countries and the uncertainty about the main drivers of influenza epidemics, it is important to point out that all countries that we examined had either the primary or the secondary peak, or both, taking place between January and March. Therefore, it seems justified to align the recommendations for the timing of the annual vaccination campaigns for all countries in this region to the Northern Hemisphere recommendations (ie late autumn), as is currently recommended by WHO^28^.

The spatial timing of influenza epidemics in the Middle East and North Africa varied greatly between seasons, both according to the longitude and to the latitude, and in most cases, there was no correspondence with what was occurring in Europe and Central Asia in the same season. A notable exception was the 2015‐2016 season, when the timing of influenza epidemics moved along an east‐to‐west longitudinal gradient in both the Middle East and North Africa (with borderline significance) and the WHO European region. This is surprising given that the typical diffusion pattern is west‐to‐east in Europe, while an opposite pattern has not been observed in this region in recent years^29^. Overall, our findings appear to suggest that the dynamics of influenza epidemics (ie where they originate, and how they spread) may be different in the Middle East and North Africa compared to Europe and Central Asia, which has implications for surveillance and influenza preparedness plans for both regions. Extending the focus to the tropical regions of Africa and Asia would help understand where influenza activity originates and the spatial timing of its activity, but a lack of suitable influenza surveillance data hampers this approach at present.

The availability of influenza surveillance data has greatly improved globally since the 2009 pandemic: this was made possible by the expansion and strengthening of the GISRS over the last fifteen years^30^ and by the sharing of virological and epidemiological data in FluNet, a repository freely accessible to influenza researchers worldwide. This has made it possible to conduct comparative studies of influenza epidemiology and seasonality in tropical and subtropical regions such as Central and southern America^5^, ^6^ and the Asia‐Pacific region^7^, ^31^. Despite these improvements, there are still some important gaps in the quality of influenza surveillance data available in the FluNet database which may be seen as limitations of the present study and should be addressed in the coming years to have a better understanding of the epidemiology of influenza in this area. No information is available in the FluNet database or the FluNet website on the national surveillance systems of the participating countries: this limits the interpretability of the results, especially the direct comparability of the data as one does not know whether the data are coming from community and/or hospital settings. However, as we were only looking at the epidemic peaks and the overall mix of viruses (for a whole season), this was probably less of an issue for our study. No data were available for Saudi Arabia, Sudan and Yemen, which are large countries with a combined population of over 100 million inhabitants. The number of seasons with data and the number of reported influenza cases (in each season and overall) varied widely across countries, and this influenced the methods used for our analysis. A number of statistical methods could have been used for the analysis, for example analysis of time series, including generalized estimating equations and seasonal ARIMA models, but we chose the epipoi software because of its ability to account for high‐ or low‐intensity years (eg by analysing the data by season, standardizing country‐ and season‐specific time series, and using of the PAF to extract timing and amplitude of peaks from standardized time series) and therefore minimize the impact of this disparity on results^6^, ^21^. In addition, the robustness of results on the timing of influenza epidemics may be somewhat less strong for countries that provided data for only four (Bahrain, Iraq) or five (Morocco) seasons, compared to countries for which data were available for all of the six seasons. The number of points in our spatial analysis was limited (also because of a lack of data for some countries, such as Libya, Sudan and Saudi Arabia) in relation to the very large longitudinal extension of this region: these made the β coefficients for the season‐specific gradients of timing sensitive to the influence of a few points. In some countries, the population is unevenly distributed throughout the territory and concentrated in well‐defined areas (eg coastal regions for countries of North Africa), but for some other countries, it would be helpful to have surveillance data at regional level, which is, however, not available in the FluNet at present.

Unlike influenza A, only a very small percentage of influenza B cases were characterized. It is usually very challenging to make reliable predictions on which lineage (Victoria or Yamagata) will dominate each influenza season, and the two lineages also differ in terms of their demographic characteristics^9^, ^32^, ^33^, ^34^, ^35^. A better knowledge of the patterns of influenza B strain circulation may be helpful to estimate the effectiveness of vaccination campaigns and optimize influenza prevention interventions. Overall, completeness of information within the FluNet database could still be improved; in addition, data validity is uncertain, as no validation with an external source of data has been carried out to date.

In conclusion, we found that the epidemiology of influenza is substantially uniform in countries of the Middle East and North Africa. In particular, the timing of influenza epidemics does not differ significantly between countries, with the partial exception of a couple of countries in the Arabian Peninsula. Influenza prevention may not be a priority at present in all countries in this area because of wars and persistent political instability. However, countries of the Middle East and North Africa are geographically contiguous and culturally interconnected, so it would be desirable that, as far as possible, influenza surveillance, preparedness and prevention activities are coordinated at a supranational level (eg regional) and implemented without disparities. According to WHO^11^, fourteen of the seventeen countries that we analysed implement seasonal influenza vaccination campaign: our findings confirm that the optimal time to vaccinate is similar to Europe and North America, that is the pre‐winter Northern Hemisphere months. The scientific evidence resulting from our regional analysis complements the one obtained from individual countries^13^, ^14^, ^15^, ^16^, ^17^; our analysis could be further enhanced by strengthening influenza surveillance networks in this region and expanding the FluNet surveillance system to neighbouring countries which currently do not report.

## CONFLICT OF INTERESTS

Clotilde El‐Guerche Séblain and Meral A. Ciblak are employees of Sanofi Pasteur. Clotilde El‐Guerche Séblain is the coordinator at Sanofi Pasteur of the research project, and she helped define the study objectives. Clotilde El‐Guerche Séblain and Meral A. Ciblak have critically revised the manuscript. When reviewing the manuscript, the revisions concerned the epidemiological findings of the study and not the public health findings or conclusions. All the other authors declare they have no conflict of interests to disclose.

## AUTHORS’ CONTRIBUTIONS

SC and JP conceived the study. SC performed the statistical analysis. SC and JP interpreted the results and wrote the first draft of the manuscript. All authors critically revised the manuscript and approved its final version.
