# Supplementary material for: Epidemiology of seasonal influenza in the Middle East and North Africa regions, 2010‐2016: Circulating influenza A and B viruses and spatial timing of epidemics
Source: Influenza Other Respir Viruses. 2018 Feb 19;12(3):344–52. doi: 10.1111/irv.12544 (PMC5907816; doi:10.1111/irv.12544)
Supplement: Supplementary file 1 [file IRV-12-344-s001.docx]

**Supplementary Table 1**. Laboratory-confirmed influenza cases reported in each country and season, by virus type, subtype and lineage. The WHO FluNet database, 2010-2016.

| **Country** | **Population (million)** | **Latitude ^(a)^** | **Longitude ^(a)^** | **Season ^(b)^** | **Influenza cases** | **% A** | **% B** | **A(H3N2)** | **2009 pandemic A(H1N1)** | **A other/not subtyped** | **B Victoria** | **B Yamagata** | **B not characterized** |
| --- | --- | --- | --- | --- | --- | --- | --- | --- | --- | --- | --- | --- | --- |
| Afghanistan | 33.3 | 33° N | 65° E | 2015-2016 | 74 | 91.9% | 8.1% | 13 | 6 | 49 | 0 | 0 | 6 |
| Algeria | 40.3 | 28° N | 3° E | 2010-2011 | 545 | 45.0% | 55.0% | 10 | 235 | 0 | 2 | 5 | 293 |
| Algeria | 40.3 | 28° N | 3° E | 2011-2012 | 143 | 97.9% | 2.1% | 139 | 1 | 0 | 0 | 3 | 0 |
| Algeria | 40.3 | 28° N | 3° E | 2012-2013 | 400 | 54.5% | 45.5% | 68 | 150 | 0 | 14 | 15 | 153 |
| Algeria | 40.3 | 28° N | 3° E | 2013-2014 | 120 | 94.2% | 5.8% | 107 | 6 | 0 | 0 | 1 | 6 |
| Algeria | 40.3 | 28° N | 3° E | 2014-2015 | 429 | 70.9% | 29.1% | 103 | 199 | 2 | 0 | 0 | 125 |
| Algeria | 40.3 | 28° N | 3° E | 2015-2016 | 195 | 99.5% | 0.5% | 76 | 118 | 0 | 0 | 0 | 1 |
| Armenia | 3.1 | 40° N | 45° E | 2015-2016 | 632 | 100.0% | 0.0% | 0 | 627 | 5 | 0 | 0 | 0 |
| Bahrain | 1.4 | 26° N | 50° E | 2012-2013 | 119 | 71.4% | 28.6% | 17 | 59 | 9 | 0 | 5 | 29 |
| Bahrain | 1.4 | 26° N | 50° E | 2013-2014 | 96 | 77.1% | 22.9% | 34 | 40 | 0 | 0 | 19 | 3 |
| Bahrain | 1.4 | 26° N | 50° E | 2014-2015 | 163 | 75.5% | 24.5% | 35 | 76 | 12 | 3 | 33 | 4 |
| Bahrain | 1.4 | 26° N | 50° E | 2015-2016 | 644 | 88.8% | 11.2% | 0 | 519 | 53 | 14 | 2 | 56 |
| Egypt | 94.7 | 27° N | 30° E | 2010-2011 | 2439 | 96.4% | 3.6% | 3 | 2193 | 156 | 8 | 8 | 71 |
| Egypt | 94.7 | 27° N | 30° E | 2011-2012 | 526 | 70.3% | 29.7% | 350 | 2 | 18 | 0 | 0 | 156 |
| Egypt | 94.7 | 27° N | 30° E | 2012-2013 | 928 | 70.5% | 29.5% | 511 | 139 | 4 | 0 | 2 | 272 |
| Egypt | 94.7 | 27° N | 30° E | 2013-2014 | 2058 | 77.3% | 22.7% | 338 | 1249 | 4 | 0 | 0 | 467 |
| Egypt | 94.7 | 27° N | 30° E | 2014-2015 | 1963 | 62.0% | 38.0% | 785 | 192 | 241 | 1 | 1 | 743 |
| Egypt | 94.7 | 27° N | 30° E | 2015-2016 | 2083 | 77.1% | 22.9% | 59 | 1522 | 26 | 1 | 0 | 475 |
| Georgia | 4.9 | 42° N | 43° E | 2010-2011 | 917 | 54.5% | 45.5% | 1 | 485 | 14 | 0 | 0 | 417 |
| Georgia | 4.9 | 42° N | 43° E | 2011-2012 | 173 | 92.5% | 7.5% | 138 | 22 | 0 | 0 | 0 | 13 |
| Georgia | 4.9 | 42° N | 43° E | 2012-2013 | 256 | 68.8% | 31.3% | 0 | 176 | 0 | 0 | 0 | 80 |
| Georgia | 4.9 | 42° N | 43° E | 2013-2014 | 129 | 96.1% | 3.9% | 122 | 2 | 0 | 0 | 0 | 5 |
| Georgia | 4.9 | 42° N | 43° E | 2014-2015 | 134 | 14.9% | 85.1% | 8 | 11 | 1 | 0 | 0 | 114 |
| Georgia | 4.9 | 42° N | 43° E | 2015-2016 | 285 | 95.4% | 4.6% | 36 | 236 | 0 | 13 | 0 | 0 |
| Iran | 82.8 | 32° N | 53° E | 2010-2011 | 1811 | 44.0% | 56.0% | 24 | 773 | 0 | 0 | 0 | 1014 |
| Iran | 82.8 | 32° N | 53° E | 2011-2012 | 973 | 90.5% | 9.5% | 875 | 2 | 4 | 0 | 0 | 92 |
| Iran | 82.8 | 32° N | 53° E | 2012-2013 | 366 | 55.5% | 44.5% | 14 | 189 | 0 | 0 | 0 | 163 |
| Iran | 82.8 | 32° N | 53° E | 2013-2014 | 754 | 70.8% | 29.2% | 417 | 117 | 0 | 0 | 0 | 220 |
| Iran | 82.8 | 32° N | 53° E | 2014-2015 | 1230 | 64.1% | 35.9% | 399 | 390 | 0 | 0 | 0 | 441 |
| Iran | 82.8 | 32° N | 53° E | 2015-2016 | 3181 | 90.4% | 9.6% | 164 | 2713 | 0 | 0 | 0 | 304 |
| Iraq | 38.1 | 33° N | 44° E | 2010-2011 | 294 | 100.0% | 0.0% | 0 | 175 | 119 | 0 | 0 | 0 |
| Iraq | 38.1 | 33° N | 44° E | 2012-2013 | 799 | 100.0% | 0.0% | 0 | 799 | 0 | 0 | 0 | 0 |
| Iraq | 38.1 | 33° N | 44° E | 2014-2015 | 299 | 96.3% | 3.7% | 29 | 20 | 239 | 0 | 0 | 11 |
| Iraq | 38.1 | 33° N | 44° E | 2015-2016 | 280 | 77.1% | 22.9% | 7 | 196 | 13 | 0 | 0 | 64 |
| Israel | 8.2 | 31° N | 34° E | 2010-2011 | 1545 | 76.5% | 23.5% | 302 | 457 | 423 | 0 | 0 | 363 |
| Israel | 8.2 | 31° N | 34° E | 2011-2012 | 565 | 60.4% | 39.6% | 148 | 42 | 151 | 0 | 0 | 224 |
| Israel | 8.2 | 31° N | 34° E | 2012-2013 | 1348 | 93.2% | 6.8% | 222 | 630 | 404 | 0 | 0 | 92 |
| Israel | 8.2 | 31° N | 34° E | 2013-2014 | 1086 | 68.9% | 31.1% | 237 | 176 | 335 | 0 | 0 | 338 |
| Israel | 8.2 | 31° N | 34° E | 2014-2015 | 862 | 87.0% | 13.0% | 251 | 71 | 428 | 0 | 0 | 112 |
| Israel | 8.2 | 31° N | 34° E | 2015-2016 | 1675 | 53.6% | 46.4% | 8 | 837 | 52 | 0 | 0 | 778 |
| Jordan | 8.2 | 31° N | 36° E | 2010-2011 | 148 | 38.5% | 61.5% | 42 | 15 | 0 | 45 | 36 | 10 |
| Jordan | 8.2 | 31° N | 36° E | 2011-2012 | 115 | 99.1% | 0.9% | 113 | 1 | 0 | 0 | 0 | 1 |
| Jordan | 8.2 | 31° N | 36° E | 2012-2013 | 230 | 48.7% | 51.3% | 7 | 105 | 0 | 41 | 25 | 52 |
| Jordan | 8.2 | 31° N | 36° E | 2013-2014 | 145 | 90.3% | 9.7% | 94 | 36 | 1 | 0 | 0 | 14 |
| Jordan | 8.2 | 31° N | 36° E | 2014-2015 | 579 | 84.1% | 15.9% | 92 | 394 | 1 | 0 | 0 | 92 |
| Jordan | 8.2 | 31° N | 36° E | 2015-2016 | 468 | 88.5% | 11.5% | 34 | 378 | 2 | 11 | 0 | 43 |
| Lebanon | 6.2 | 33° N | 35° E | 2014-2015 | 125 | 21.6% | 78.4% | 6 | 21 | 0 | 0 | 0 | 98 |
| Lebanon | 6.2 | 33° N | 35° E | 2015-2016 | 177 | 49.2% | 50.8% | 32 | 55 | 0 | 0 | 0 | 90 |
| Morocco | 33.7 | 32° N | 5° W | 2010-2011 | 313 | 60.4% | 39.6% | 50 | 139 | 0 | 0 | 0 | 124 |
| Morocco | 33.7 | 32° N | 5° W | 2011-2012 | 250 | 94.8% | 5.2% | 236 | 1 | 0 | 1 | 2 | 10 |
| Morocco | 33.7 | 32° N | 5° W | 2012-2013 | 145 | 35.9% | 64.1% | 2 | 50 | 0 | 27 | 14 | 52 |
| Morocco | 33.7 | 32° N | 5° W | 2014-2015 | 199 | 32.7% | 67.3% | 25 | 38 | 2 | 0 | 17 | 117 |
| Morocco | 33.7 | 32° N | 5° W | 2015-2016 | 271 | 88.9% | 11.1% | 57 | 184 | 0 | 0 | 1 | 29 |
| Oman | 3.4 | 21° N | 57° E | 2010-2011 | 729 | 70.2% | 29.8% | 15 | 307 | 190 | 0 | 0 | 217 |
| Oman | 3.4 | 21° N | 57° E | 2011-2012 | 354 | 73.7% | 26.3% | 53 | 72 | 136 | 0 | 0 | 93 |
| Oman | 3.4 | 21° N | 57° E | 2012-2013 | 205 | 73.2% | 26.8% | 36 | 43 | 71 | 0 | 0 | 55 |
| Oman | 3.4 | 21° N | 57° E | 2013-2014 | 795 | 90.1% | 9.9% | 221 | 421 | 74 | 0 | 3 | 76 |
| Oman | 3.4 | 21° N | 57° E | 2014-2015 | 1158 | 77.9% | 22.1% | 285 | 613 | 4 | 0 | 0 | 256 |
| Oman | 3.4 | 21° N | 57° E | 2015-2016 | 1780 | 60.2% | 39.8% | 339 | 730 | 2 | 0 | 73 | 636 |
| Pakistan | 202.0 | 30° N | 70° E | 2010-2011 | 833 | 89.9% | 10.1% | 8 | 434 | 307 | 0 | 0 | 84 |
| Pakistan | 202.0 | 30° N | 70° E | 2011-2012 | 450 | 84.4% | 15.6% | 125 | 105 | 150 | 1 | 1 | 68 |
| Pakistan | 202.0 | 30° N | 70° E | 2012-2013 | 222 | 50.9% | 49.1% | 75 | 30 | 8 | 47 | 38 | 24 |
| Pakistan | 202.0 | 30° N | 70° E | 2013-2014 | 253 | 51.8% | 48.2% | 48 | 83 | 0 | 18 | 18 | 86 |
| Pakistan | 202.0 | 30° N | 70° E | 2014-2015 | 75 | 70.7% | 29.3% | 31 | 21 | 1 | 0 | 0 | 22 |
| Pakistan | 202.0 | 30° N | 70° E | 2015-2016 | 440 | 84.8% | 15.2% | 82 | 291 | 0 | 0 | 16 | 51 |
| Qatar | 2.3 | 25° N | 51° E | 2010-2011 | 136 | 86.8% | 13.2% | 0 | 81 | 37 | 0 | 0 | 18 |
| Qatar | 2.3 | 25° N | 51° E | 2011-2012 | 552 | 76.4% | 23.6% | 84 | 250 | 88 | 0 | 0 | 130 |
| Qatar | 2.3 | 25° N | 51° E | 2012-2013 | 543 | 65.4% | 34.6% | 0 | 173 | 182 | 0 | 1 | 187 |
| Qatar | 2.3 | 25° N | 51° E | 2013-2014 | 3401 | 78.9% | 21.1% | 0 | 1367 | 1315 | 0 | 0 | 719 |
| Qatar | 2.3 | 25° N | 51° E | 2014-2015 | 2017 | 75.1% | 24.9% | 0 | 666 | 848 | 0 | 0 | 503 |
| Qatar | 2.3 | 25° N | 51° E | 2015-2016 | 4932 | 81.7% | 18.3% | 0 | 2683 | 1345 | 0 | 0 | 904 |
| Tunisia | 11.1 | 34° N | 9° E | 2010-2011 | 216 | 72.7% | 27.3% | 6 | 150 | 1 | 0 | 0 | 59 |
| Tunisia | 11.1 | 34° N | 9° E | 2011-2012 | 76 | 88.2% | 11.8% | 67 | 0 | 0 | 0 | 0 | 9 |
| Tunisia | 11.1 | 34° N | 9° E | 2012-2013 | 387 | 58.1% | 41.9% | 23 | 202 | 0 | 0 | 38 | 124 |
| Tunisia | 11.1 | 34° N | 9° E | 2013-2014 | 75 | 92.0% | 8.0% | 64 | 5 | 0 | 0 | 0 | 6 |
| Tunisia | 11.1 | 34° N | 9° E | 2014-2015 | 273 | 56.0% | 44.0% | 38 | 115 | 0 | 0 | 21 | 99 |
| Tunisia | 11.1 | 34° N | 9° E | 2015-2016 | 435 | 95.9% | 4.1% | 183 | 234 | 0 | 0 | 18 | 0 |
| Turkey | 80.3 | 39° N | 35° E | 2010-2011 | 1396 | 49.3% | 50.7% | 250 | 438 | 0 | 0 | 0 | 708 |
| Turkey | 80.3 | 39° N | 35° E | 2011-2012 | 848 | 72.4% | 27.6% | 597 | 2 | 15 | 89 | 2 | 143 |
| Turkey | 80.3 | 39° N | 35° E | 2012-2013 | 1014 | 97.2% | 2.8% | 53 | 930 | 3 | 0 | 0 | 28 |
| Turkey | 80.3 | 39° N | 35° E | 2013-2014 | 1056 | 79.5% | 20.5% | 814 | 24 | 2 | 0 | 0 | 216 |
| Turkey | 80.3 | 39° N | 35° E | 2014-2015 | 1973 | 49.2% | 50.8% | 181 | 783 | 6 | 0 | 0 | 1003 |
| Turkey | 80.3 | 39° N | 35° E | 2015-2016 | 8224 | 84.6% | 15.4% | 2330 | 4615 | 14 | 0 | 0 | 1265 |
| **Total** | **654.0** | **21° N - 42° N** | **5° W - 70° E** | **83** | **70,532** | **75.9%** | **24.1%** | **12,848** | **33,137** | **7,567** | **336** | **418** | **16,226** |

^(a)^ Latitude of the country centroid (if available) or largest city.

^(b)^ A season was defined as the period between July 1^st^ and June 30^th^ of next year. Only seasons with 50 or more reported influenza cases and 20 or more weeks of data reporting were included.
