# Supplementary material for: Epidemiology of seasonal influenza in the Middle East and North Africa regions, 2010‐2016: Circulating influenza A and B viruses and spatial timing of epidemics
Source: Influenza Other Respir Viruses. 2018 Feb 19;12(3):344–52. doi: 10.1111/irv.12544 (PMC5907816; doi:10.1111/irv.12544)
Supplement: Supplementary file 2 [file IRV-12-344-s002.docx]

**Supplementary document 1**. Standardized time-series of laboratory-confirmed influenza (from season 2010-2011 to 2015-2016; blue line) and periodic annual function (red line) for countries in Middle East and North Africa included in the analysis (see text for detail). Please refer to the text for details on methods.
